# OMPB: An Omnidirectional-Mobile Paddle Boat Designed for Narrow Water Areas

**DOI:** 10.3390/s26030866

**Published:** 2026-01-28

**Authors:** Zhangze Gan, Ziye Huang, Bin Deng, Huangyu Gong

**Affiliations:** 1 School of Hydraulic and Ocean Engineering, Changsha University of Science and Technology, Changsha 410114, China; zhangzegump@csust.edu.cn (Z.G.); 202304330121@csust.edu.cn (H.G.); 2 Elite Engineering School, Changsha University of Science and Technology, Changsha 410114, China; 3 School of Electrical and Information Engineering, Changsha University of Science and Technology, Changsha 410114, China; ziye06csust@163.com; 4 Hunan Provincial Engineering Technology Research Center for Smart Water Resources Digital Twin, Hunan Water Resources and Hydropower Survey, Design, Planning and Research Co., Ltd., Changsha 410007, China

**Keywords:** omnidirectional movement, paddle boat, trajectory tracking, kinematics, dynamics

## Abstract

This paper presents the design of an omnidirectional-mobile paddle boat (OMPB) used in narrow rivers, ponds, and canals. Compared with common propeller boats, the OMPB has advantages such as zero turning radius and shallow draft. Firstly, a prototype is built in which there are four paddles connected with four DC motors, allowing the boat to move like an omnidirectional Mecanum-wheeled vehicle. Subsequently, to develop the OMPB’s autonomous navigation algorithms, a kinematic model is established and dynamic analysis is performed. To improve the ability of resisting disturbances and control precision, a control algorithm based on fuzzy controller is designed for trajectory tracking. Experimental validations cover trajectory tracking performance during both straight-line navigation and turning maneuvers. The results demonstrate that the OMPB is competent to carry out omnidirectional movement, and the actual navigation trajectory is highly consistent with the theoretical trajectory, with a tracking error within 40 mm and a heading angle error within 1.8°. The OMPB platform can be reformed into special-purpose vessels for floating garbage collection and fish feeding in narrow water areas.

## 1. Introduction

Ships and boats are commonly used as overwater production tools [1]. With advancements in intelligent technologies, there is a growing demand for surface vehicles in various civilian sectors [2,3,4]. As examples that have been researched and developed, there are surface vehicles for fish farming and feed distribution [5,6], floating plastic garbage collection with integrated intelligent systems [7], aquatic plants harvesting [8], and maritime unmanned vessels [9]. Compared with surface vehicles employed in the ocean, their use in inland rivers is closer to human life and has greater potential application value [10].

Surface vehicles rely on propulsion systems to generate thrust for movement. Power propulsion includes propellers, water-jet propulsion, bionic structure propulsion, and paddle wheels. Surface vehicles typically utilize propellers to provide thrust and rudder generation to achieve steering, and scholars have conducted extensive research on such propulsion systems [11,12,13]. Among propeller propulsion systems, the Azipod thruster is a type of 360° rotatable podded propulsion system which can reduce the turning radius through the overall rotation of the pod [14]. Since the propeller is typically mounted at the stern, an Azipod-equipped vessel will experience bow deflection during sway movement. In contrast, the OMPB system enables direct sway movement without altering the vessel’s heading. In addition, the intricate mechanical structure of the Azipod thruster leads to high manufacturing costs and maintenance difficulties, and the propeller blades are prone to harming underwater organisms or becoming entangled with submerged vegetation and debris. Water-jet propelled vessels also have a large turning radius and face greater control difficulties [15]. Bionic propulsion systems, inspired by seabirds, beavers, and other organisms, can achieve highly flexible steering [16,17]. However, their intricate propulsion structures require sophisticated control algorithms for stable operation, and they are also prone to entanglement with underwater vegetation and debris, which impairs propulsion efficiency and equipment safety. These factors, therefore, make the existing surface vehicles particularly unsuitable for small, narrow water areas [5].

Surface vehicles equipped with paddle wheel propulsion systems usually generate thrust by propelling water through the blades on the underwater section of the paddle wheel, while the portion of the blade above the water surface performs no useful work. These movement characteristics and structural design result in lower efficiency and slower speeds for paddle wheel-driven vessels. However, they also offer advantages such as shallow draft and reduced susceptibility to entanglement. As a result, paddle wheel vessels are well-suited for operations in small, shallow water areas such as rivers, ponds, urban waterways, and narrow channels, including tasks like floating garbage cleaning and fish feed distribution [6].

Numerous scholars have conducted fruitful research on omnidirectional-mobile vessels. Gao et al. developed a small dual-hull unmanned vessel driven by propellers. This vessel utilizes a detailed analysis of its kinematic model to develop autonomous navigation algorithms, but it requires a larger turning radius during maneuvers [18]. Tadakuma et al. developed the Omni-paddle, an amphibious spherical rotary paddle mechanism with omnidirectional mobility. However, its hydrodynamic performance during water navigation is inferior to that of a normal paddle [19]. Chengzhi Ruan et al. designed a river crab automatic feed delivery vessel driven by dual-paddle wheels, which achieved flexible navigation and reduced turning radius. Additionally, due to the nonlinear characteristics of vessel movement, the authors introduced a fuzzy PID dual-loop control algorithm to improve the system’s positioning accuracy. However, the control objects in this case are the two paddle wheels [20]. Current research indicates that existing typical vessels are not efficient in achieving small-radius turns, omnidirectional movement, and maneuverability in narrow water areas.

The navigation direction of a dual-paddle boat is steered by the autonomous propulsion generated by the left and right paddle wheels. The movement direction and steering control of a dual-paddle boat are determined by the differential thrust and direction of the left and right paddle wheels. However, due to the distance between the two paddle wheels on the hull, a certain turning radius is still required, which can limit the vessel’s ability to efficiently cover narrow corners of water areas. Additionally, the action of water currents often leads to the accumulation of garbage in these narrow corners [21]. According to the above situation, designing a small vessel platform capable of agile movement in narrow waterways is of great importance. Therefore, this paper proposes a paddle boat with omnidirectional movement capabilities, OMPB, enabling flexible navigation in small and narrow water areas.

**Figure 1:**
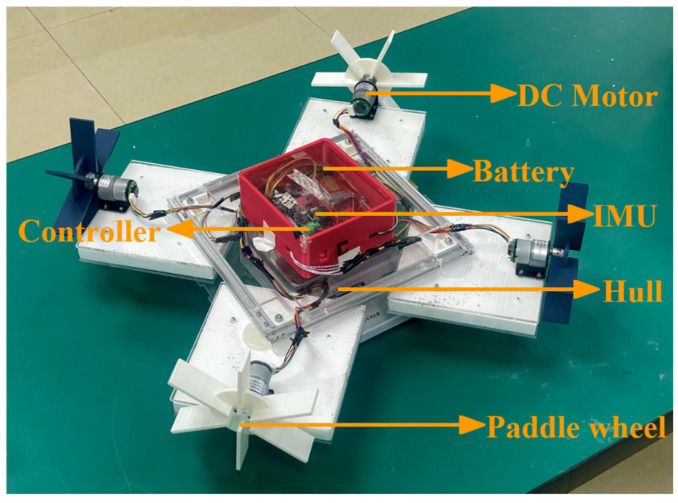
Configuration of OMPB.

This paper presents the design of an omnidirectional-mobile paddle boat based on four paddle wheels connected with four DC motors which are centrally symmetric (Figure 1). In comparison to propeller-driven and dual-paddle boats, the OMPB offers advantages such as omnidirectional movement, zero turning radius, and shallow draft. The OMPB platform can be reformed into special-purpose vessels for collecting floating garbage and feeding fish feed in small narrow water areas. Notably, controlling the rotational speed of the four paddle wheels in an omnidirectional vessel is more complex than controlling two paddle wheels, making the study of control methods for four paddle wheels of great importance.

**Figure 2:**
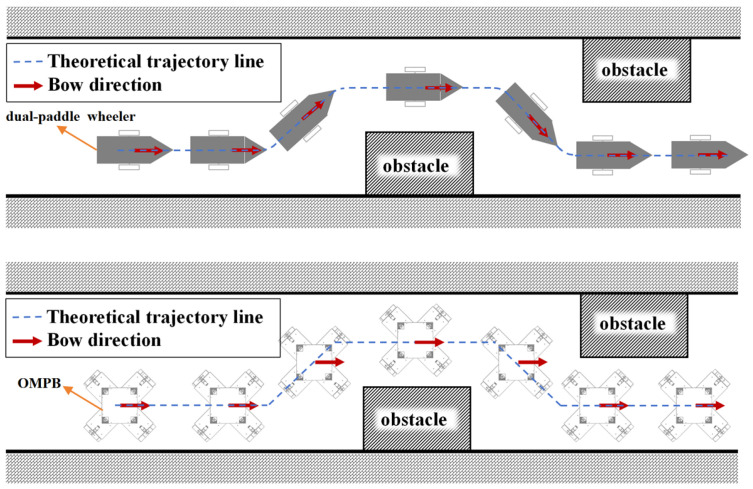
Comparison of obstacle-avoiding trajectories between dual-paddle boat and OMPB.

The design of the OMPB faces three key challenges compared to conventional vessel designs. Structural Innovation: Currently, traditional vessel structures often employ differential drive with two horizontal propellers [22], which makes it challenging to achieve a small or even zero turning radius. Therefore, innovative improvements are needed in vessel structures. It can be seen from Figure 2 that the greatest advantage of the OMPB we designed lies in its capability to turn or avoid obstacles without changing the bow direction. Kinematics and Dynamic Analysis: The structure of the OMPB is unique, and currently, there is limited research on it. To better understand and control the vessel’s motion behavior, it is necessary to establish the kinematic model of the OMPB and conduct dynamic analysis. Control System Complexity: The OMPB needs to deal with the increased complexity of the control system due to the integration of multiple paddle wheels and the requirement for achieving coordinated movement.

The organization of the remaining paper is as follows. Section 2 provides an overview of the structural design of the omnidirectional-mobile paddle boat. Section 3 establishes the kinematic model of the OMPB and conducts dynamic analysis. Section 4 introduces the fuzzy adaptive PID control algorithm for the four paddle wheels. Experimental results and analysis are presented in Section 5 Finally, Section 6 provides a conclusion for the paper.

## 2. Prototype Design of OMPB

To verify the omnidirectional movement performance of the OMPB, a small-sized omnidirectional-mobile paddle boat (Figure 1) was designed for experiments. The four-wheel vessel consists of a hull, battery, paddle wheels, DC motors with encoders, control board, Inertial Measurement Unit (IMU), and electronic control box.

The hull is composed of four square floating blocks, which are fixed by a square aluminum frame and distributed in a central-symmetric pattern, and the overall dimensions l×b×h=600 mm×600 mm×120 mm. Then, the radius (R) and width (S) of the paddle wheels are 95 mm and 35 mm, and the water intrusion depths of the vessel and four paddle wheels are d = 25 mm and c = 60 mm, respectively. Four paddle wheels are symmetrically mounted on the square floating blocks, with adjacent paddle wheels perpendicular to each other, and each paddle wheel is controlled by an independent DC motor and driven through a transmission mechanism. The electronic control box is installed in the center of the hull, housing control circuit boards and an Inertial Measurement Unit (IMU).

## 3. Model Description

### 3.1. Kinematic Model of OMPB

**Figure 3:**
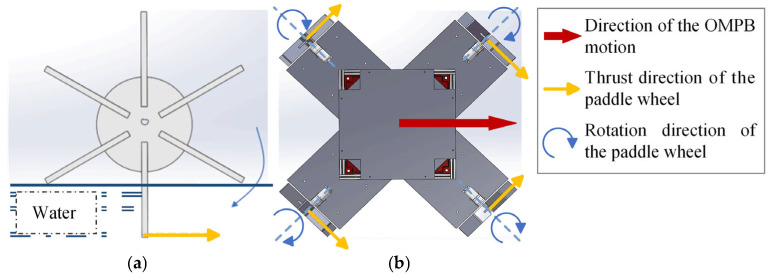
Motion illustration of OMPB: (**a**) thrust direction of single paddle wheel; (**b**) rightward motion of OMPB.

When a paddle wheel rotates in water, the thrust force generated is the same regardless of rotation direction under identical conditions. The thrust force on an individual wheel is shown in Figure 3a, and it has similar characteristics to the Mecanum wheel. By controlling the direction and speed of the four wheels, the OMPB can achieve movement in all directions, including surge, sway, diagonal, and rotational movements. An illustration of rightward movement is shown in Figure 3b.

**Figure 4:**
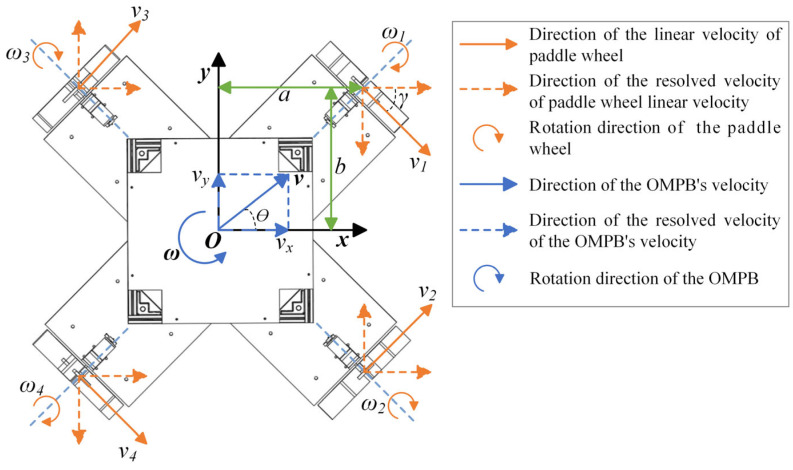
The kinematic vector diagram of the OMPB.

When a vessel navigates rapidly on the water surface, it has six degrees of freedom: translation (surge, sway, and heave) and rotation (roll, pitch, and yaw). Notably, vessel navigation is susceptible to environmental disturbances. As a result, its kinematic mathematical model becomes extremely complex. To simplify the initial kinematic modeling, we first assume that the OMPB moves slowly in a static water area to establish the basic motion relationship, and only consider three degrees of freedom: surge, sway, and yaw. However, the subsequent dynamic analysis and fuzzy PID control algorithm fully consider environmental disturbances and dynamic changes, enabling adaptation to non-static application scenarios. Taking inspiration from the kinematic model of a Mecanum-wheeled robot [23,24], a motion vector diagram of the OMPB is established as shown in Figure 4.

In the kinematic vector diagram shown in Figure 4, the parameters are defined as follows:

(1) Oxy represents the motion coordinate system, where O is the geometric center of the vessel. The x-axis always points towards the transverse axis of the vessel, and the y-axis is perpendicular to the x-axis.
(2) a and b represent the components of the vector from the vessel’s center O to the center of each paddle wheel, in the x-axis and y-axis directions, respectively, where $a_{i}=\left\{a,a,-a,-a\right\}$, $b_{i}=\left\{b,-b,b,-b\right\}$, and i=1,2,3,4.
(3) θ represents the heading angle of the vessel during motion.
(4) v represents the velocity of the vessel, with $v_{x}$ and $v_{y}$ representing its components along the x-axis and y-axis, respectively. It is worth noting that $v_{x}=v\cos\theta$, $v_{y}=v\sin\theta$. $v_{i}$ is the linear velocity of the i-th paddle wheel.
(5) ω represents the angular velocity of the vessel’s motion, positive in the counterclockwise direction. $\omega_{i}$ is the angular velocity of the i-th paddle wheel.
(6) γ represents the angle between the velocity vector v on the paddle wheel and the x-axis of the vessel. Here, $\gamma_{i}=\left\{-45{}^{\circ},45{}^{\circ},45{}^{\circ},-45{}^{\circ}\right\}$, where i=1,2,3,4.
(7) $R_{i}$ represents the radius of the *i*-th paddle wheel, where $R_{1}=R_{2}=R_{3}=R_{4}=R$.

The relationship between the linear velocities of the paddle wheels and the vessel’s velocity is derived as follows:

(1) $$\left\{\begin{array}{l} v_{i}\;\times \;\cos(\gamma_{i})\;={\;v}_{x}-\omega \;\times \;b_{i}\\ -v_{i}\;\times \;\sin(\gamma_{i})\;=\;v_{y}\;+\;\omega \;\times \;a_{i} \end{array}\right.,\;(i\;=\;1,\;2,\;3,\;4)$$

The equation can be derived as follows:

(2) $$\left\{\begin{array}{l} v_{i}\;=\;\frac{v_{x}\;+\;v_{y}\;+\;\omega \;\times \;(a_{i}-b_{i})}{\cos(\gamma_{i})\;-\sin(\gamma_{i})},\;(i\;=\;1,\;4)\\ v_{i}\;=\;\frac{v_{x}-v_{y}-\omega \;\times \;(a_{i}\;+{\;b}_{i})}{\cos(\gamma_{i})\;+\;\sin(\gamma_{i})},\;(i\;=\;2,\;3) \end{array}\right.,$$

Therefore:

(3) $$\left\{\begin{array}{l} v_{1}\;=\;\frac{\sqrt{2}}{2}(v_{x}\;+\;v_{y}\;+\;\omega \;\times \;(a-b))\\ v_{2}\;=\;\frac{\sqrt{2}}{2}(v_{x}-v_{y}-\omega \;\times \;(a-b))\\ v_{3}\;=\;\frac{\sqrt{2}}{2}(v_{x\;}-\;v_{y}-\omega \;\times \;(-a\;+\;b))\\ v_{4}\;=\;\frac{\sqrt{2}}{2}(v_{x}\;+\;v_{y}\;+\;\omega \;\times \;(-a\;+\;b)) \end{array}\right.,$$

where $v_{i}=\omega_{i}\times R_{i}=\omega_{i}\times R$; the expression can be transformed into an angular velocity form:

(4) $$\left\{\begin{array}{c} \left[\begin{array}{c} \omega_{1}\\ \omega_{2}\\ \omega_{3}\\ \omega_{4} \end{array}\right]\;=\;\frac{\sqrt{2}}{2R}\left[\begin{array}{ccc} \begin{array}{c} 1\\ 1\\ 1\\ 1 \end{array} & \begin{array}{c} 1\\ -1\\ -1\\ 1 \end{array} & \begin{array}{c} a-b\\ b-a\\ a-b\\ b-a \end{array} \end{array}\right]\left[\begin{array}{c} v_{x}\\ v_{y}\\ \omega \end{array}\right],\;\left[\begin{array}{c} v_{x}\\ v_{y}\\ \omega \end{array}\right]\text{≠}\left[\begin{array}{c} 0\\ 0\\ 0 \end{array}\right]\\ \;\left[\begin{array}{c} \omega_{1}\\ \omega_{2}\\ \omega_{3}\\ \omega_{4} \end{array}\right]\;=\;\left[\begin{array}{c} 0\\ 0\\ 0\\ 0 \end{array}\right],\;\left[\begin{array}{c} v_{x}\\ v_{y}\\ \omega \end{array}\right]=\left[\begin{array}{c} 0\\ 0\\ 0 \end{array}\right] \end{array}\;\right.,$$

Equation (4) represents the relationship between the velocity of the vessel and the angular velocity of the paddle wheels. When ${\left[v_{x}\;{\;v}_{y}\;\omega \right]}^{T}\neq {\left[0\;0\;0\right]}^{T}$, according to this equation, the OMPB can achieve omnidirectional movement on the water surface by appropriately setting the angular velocities of the four paddle wheels. The angular velocities of the four paddle wheels can be obtained from the velocities of the OMPB ($v_{x}$ and $v_{y}$) and the yaw angle. Additionally, by using the motor speed feedback from the controlled paddle wheels, the angular velocity of the OMPB can be determined, ultimately enabling precise closed-loop control of the control system.

### 3.2. Dynamic Analysis of OMPB

The paddle wheels serve as the propulsion source for the OMPB. When the paddle wheels rotate, they generate a disturbance on the water surface, providing thrust force and lift force to the vessel. Dynamic analysis of the OMPB is essential for establishing simulation models and controlling vessel motion. Hong [6] and Yang [25] have conducted detailed analyses of the driving forces generated by individual wheels and have provided corresponding calculation equations.

The horizontal thrust and vertical lift of an individual wheel are represented by Equation (5):

(5) $$\left\{\begin{array}{ll} T_{d}= & \frac{1}{8}\rho \pi c^{2}\mathrm{Bs}(\frac{1}{4}\omega R\cos\varphi \;-\;v_{0})K_{T}\\ & L_{d}\;=\;\frac{1}{32}\rho \pi c^{2}\mathrm{Bs}\omega RK_{L}\sin\varphi \end{array}\right.,$$

where $T_{d}$ is the thrust of a single paddle wheel, $\left[N\right]$; ρ is the density of water, [$\mathrm{kg}{\cdot m}^{-3}]$; c is the immersion depth of the paddle wheel blade in the water, $\left[m\right]$; B is the number of paddle wheel blades; s represents the width of the paddle wheel blades, $\left[m\right]$; ω is the angular velocity of the paddle wheel, $\left[r\cdot s^{-1}\right]$; R is the paddle wheel radius, $\left[m\right]$; φ is the immersion angle of the paddle wheel blades; $v_{0}$ is the initial speed of the paddle wheels, $\left[m\cdot s^{-1}\right]$; $K_{T}$ is the thrust coefficient; $K_{L}$ is the lift coefficient. The thrust coefficient $K_{T}$ and lift coefficient $K_{L}$ are obtained through paddle wheel water towing experiments.

From Equation (5), under identical conditions, the magnitude and direction of the thrust force of a single paddle wheel can be adjusted by varying its angular velocity ω.

**Figure 5:**
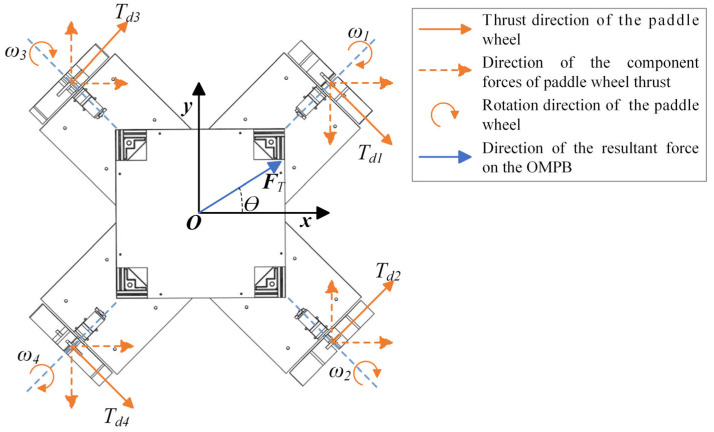
Force analysis diagram of OMPB.

The four paddle wheels on the OMPB are symmetrically distributed around the center and perpendicular to each other (as shown in Figure 3b). The analysis diagram of the thrust force exerted on these wheels is illustrated in Figure 5.

From this, the total thrust $F_{T}$ of the vessel can be derived as shown in Equation (6).

(6) $$\left\{\begin{array}{c} F_{T}\sin\theta \;=\sum_{i=1}^{4}T_{\mathrm{di}}\sin\gamma_{i}\\ F_{T}\cos\theta \;=\sum_{i=1}^{4}T_{\mathrm{di}}\cos\gamma_{i} \end{array}\right.,$$

where $F_{T}$ is the resultant force of the OMPB; $T_{d}$ is the thrust force of the i-th paddle wheel; γ is the angle between the velocity vector v on the paddle wheel and the axis on the vessel, $\gamma_{i}=\left\{-45{}^{\circ},45{}^{\circ},45{}^{\circ},45{}^{\circ}\right\}$, where i=1,2,3,4.

From Equation (6), it is evident that by generating thrust in different directions and magnitudes using the four wheels, the OMPB can achieve motion in lateral, longitudinal, diagonal, and rotational directions.

## 4. Omnidirectional Movement Control of OMPB

### 4.1. Fuzzy Adaptive PID Control Algorithm

PID control is a commonly used control algorithm that has been widely applied in the industrial control field. The basic equation representing the output signal *u*(*t*) of PID control is expressed as Equation (7).

(7) $$u(t)=K_{p}\left(e(t)+\frac{1}{T_{t}}\int_{0}^{t}e(t)\mathrm{dt}+{\;T}_{D}\frac{\mathrm{de}(t)}{\mathrm{dt}}\right),$$

where $K_{p}$ represents the proportional gain; $T_{t}$ and $T_{D}$ represent the integration time constant and the differential time constant, respectively; *u*(*t*) represents the output signal of the PID controller; *e*(*t*) represents the deviation between the setpoint and the measured value.

PID control has the advantages of low computational cost, ease of application, and straightforward implementation in engineering. However, when dealing with stochastic non-stationary fluctuating multivariable dynamic systems, it can be challenging to determine appropriate PID controller parameters, resulting in limited control accuracy [26].

When navigating on the water surface, the OMPB is susceptible to environmental disturbances. A single set of PID parameters cannot meet the requirements for trajectory tracking performance, and different PID parameters need to be selected at different times [27]. Recently, there have been various control algorithms available for nonlinear motion characteristics, such as genetic control, neural network control, and fuzzy control. Genetic algorithms have the advantages of adapting to nonlinearities and searching for multiple optimal solutions, but they are prone to getting trapped in local optima. Neural network control algorithms can achieve high control accuracy and support multiple outputs but require extensive pre-training. Fuzzy control algorithms, based on rich expert experience, make it easy to establish fuzzy rule bases and exhibit good robustness [28].

In the navigation process of the OMPB, the draft depth, inertia, and sailing speed are all dynamically changing in real time. Therefore, under limited resources, the fuzzy PID control algorithm is a more suitable control approach. The fuzzy PID control algorithm is designed based on a fuzzy controller, utilizing the fundamental theories and methods of fuzzy mathematics. It represents the conditions and operations of rules using fuzzy sets and stores these fuzzy control rules and related information, such as indicators and initial PID parameters, in the fuzzy knowledge base. The membership functions and fuzzy rules are primarily obtained through experimental experience. Then, the computer automatically adjusts the PID parameters based on the actual response of the control system using fuzzy inference, thereby achieving optimal adjustment.

**Figure 6:**
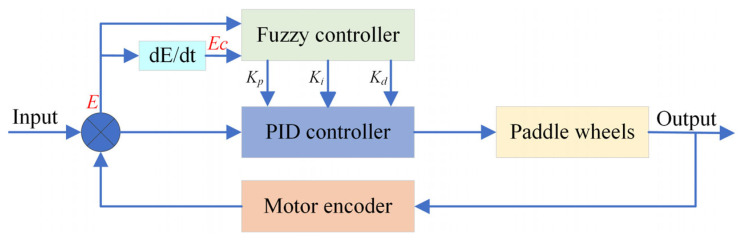
Flowchart of fuzzy PID control algorithm.

The rotational speed of each paddle wheel determines the OMPB’s navigation speed and direction. Therefore, setting the desired rotational speed of each paddle wheel as the target speed is crucial. Firstly, the current rotational speed of each paddle wheel is obtained by reading the encoder of the DC motor. This allows us to determine the deviation E between the current speed and the target speed, as well as the rate of change of the deviation, Ec, which is the derivative of the deviation. Then, fuzzy inference is performed based on fuzzy rules, and the fuzzy parameters are fuzzified accordingly. Finally, the fuzzy controller outputs the real-time *K_p_*, *K_i_*, and *K_d_* parameters to the PID controller, enabling precise control of the paddle wheel’s rotational speed. The overall control workflow is illustrated in Figure 6. During the operation of the control system, fuzzy control utilizes fuzzy inference to track the real-time changes in input parameter errors and adjust the PID control parameters. This approach significantly improves the accuracy of the control system.

### 4.2. Fuzzy Controller for Paddle Wheel Speed

**Figure 7:**
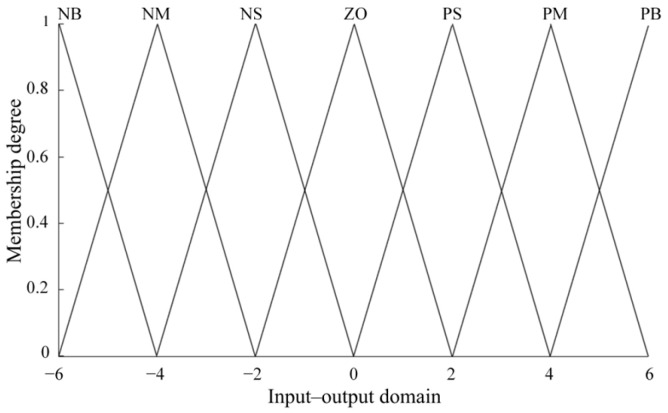
Membership functions of inputs and outputs.

The fuzzy control algorithm for the paddle wheel rotational speed of the OMPB primarily involves designing a fuzzy controller and a PID controller. The input to the fuzzy controller is the deviation *E* between the current rotational speed of the paddle wheel and the target rotational speed, as well as the rate of change of the deviation *Ec*. The fuzzy controller outputs adjusted values of *K_p_*, *K*_i_, and *K_d_*, which are then transferred to the PID controller. Through experiments conducted on the prototype, a set of relatively suitable PID parameters is determined, and the fuzzy universe of discourse for the input and output variables is set to [−6, 6]. It is represented using seven fuzzy sets (NB, NM, NS, ZO, PS, PM, and PB). The order of these sets from small to large is as follows: “NB” represents “negative big,” “NM” represents “negative medium,” “NS” represents “negative small,” “ZO” represents “zero,” “PS” represents “positive small,” “PM” represents “positive medium,” and “PB” represents “positive big.” The membership functions are shown in Figure 7, and for the sake of simplicity and highly accurate control, the input and output membership functions are uniformly distributed triangular functions with center points at −6, −4, −2, 0, 2, 4, and 6. In the design of input and output membership functions, the use of triangular membership functions can achieve better control effects by combining expert experience and fuzzy statistics. Compared to nonlinear membership functions such as Gaussian membership functions, triangular ones are simpler and computationally faster.

In the design of fuzzy rules, having too many fuzzy sets leads to complex computations, while having too few fuzzy sets may not achieve the desired control effect. Based on the universe of discourse of the input and output variables, seven fuzzy sets are considered appropriate. Fuzzy rules are the core of fuzzy control technology and are generally obtained through experiments. The values of the proportional, integral, and differential regulation parameters in the PID controller will be added to the original previous values with the values of *K_p_*, *K_i_*, and *K_d_* output from the fuzzy controller, respectively. The fuzzy rules for output variables are shown in Table 1.

### 4.3. Simulink Simulation Analysis

**Figure 8:**
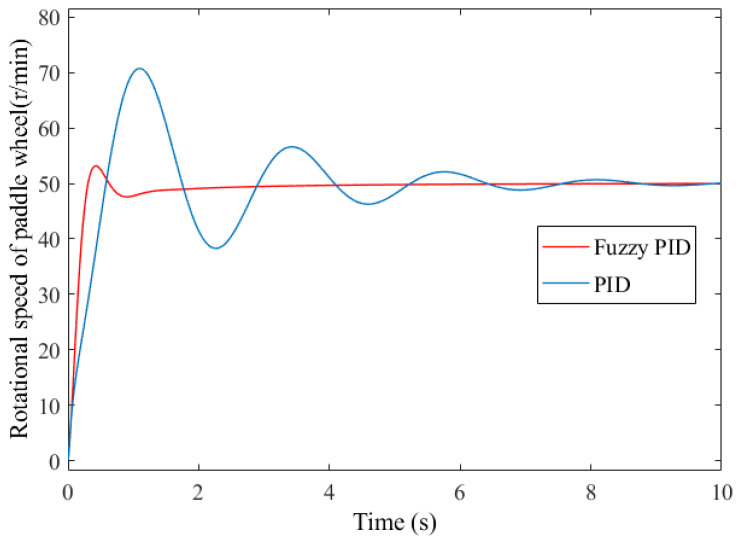
Simulation response curves of speed control of paddle wheel.

By utilizing the Simulink simulation tool, a fuzzy PID controller model for the paddle wheel of the OMPB was established. The rotational speed of the paddle wheel was set at 50 r·min^−1^ as the system input, the system operation time was configured to 10 s, and the sampling interval was set to 1.0 ms. A conventional PID controller was also developed for the purpose of control algorithm comparison. The simulation results were exported to the workspace, followed by a comparative analysis of the control performances of the two controllers. The tracking response curves of traditional PID and fuzzy PID control for rotational speed of paddle wheel regulation are illustrated in Figure 8.

As can be seen from Figure 8, in the process of speed control, with the elapse of simulation time, the response curve of fuzzy PID control becomes increasingly stable with a further reduction in fluctuation amplitude, and the overall response characteristics exhibit a more desirable smooth transition. The speed overshoot of the traditional PID controller is 41.5% with a settling time of 9.7 s, whereas the fuzzy PID controller achieves an overshoot of 6.4% and a settling time of 2.3 s. Compared with the traditional PID controller, the overshoot is reduced by 35.1% points and the settling time is shortened by 7.4 s. The fuzzy PID controller not only features a smaller overshoot, shorter transition time, and faster response speed, but can also recover stability more rapidly when subjected to system parameter variations or external disturbances. In contrast to the traditional PID control method, the deviation between its output value and the setpoint is smaller, enabling it to maintain the output more accurately near the target value. Thus, the fuzzy PID controller demonstrates remarkable advantages in the transient response characteristics of the control system.

### 4.4. Realization of Omnidirectional Movement Control

**Figure 9:**
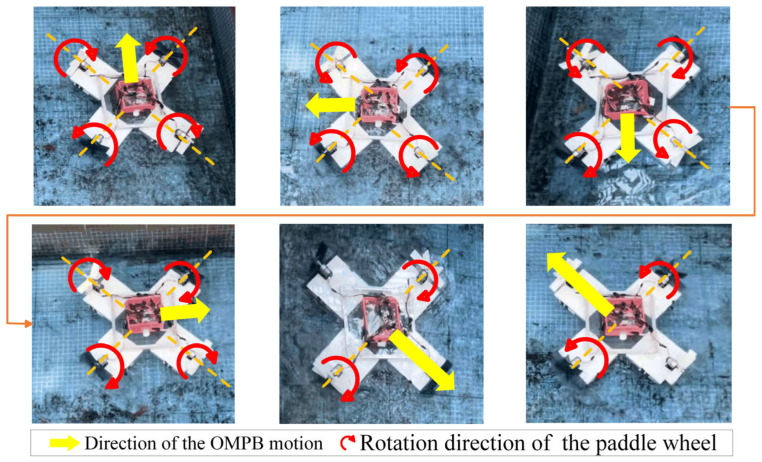
Omnidirectional motions.

The paddle wheel is connected to the DC motor through couplings and flanges. Each of the four DC motors independently controls the rotational speed and direction of each paddle wheel. The paddle wheels generate thrust by gliding on the water surface. Based on the kinematic model and dynamic analysis, it is known that by controlling the rotational direction and speed of the four paddle wheels appropriately, the thrust generated on different paddle wheels can be canceled out or superimposed in certain directions. This enables the control of the OMPB’s direction, making it move in the direction of the resultant force it experiences. Figure 9 shows the omnidirectional motions of the OMPB. As shown in the figure, it can be confirmed that the OMPB is capable of moving in any direction (forward–backward, left–right, and oblique direction).

**Figure 10:**
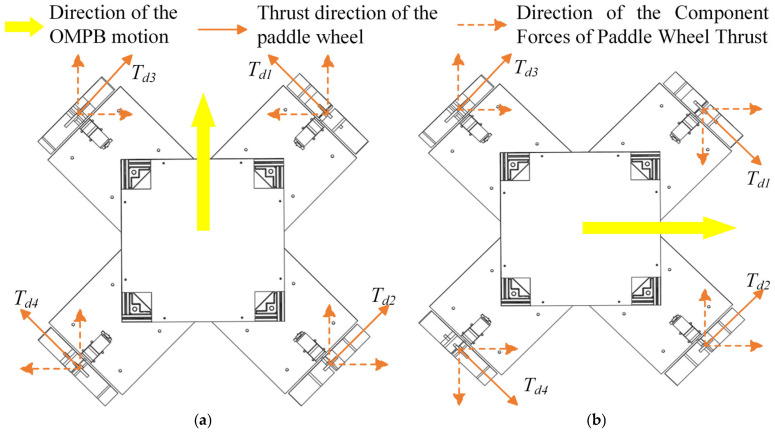

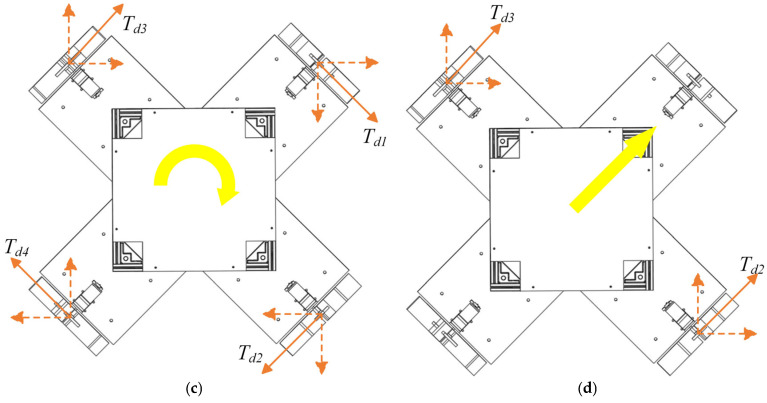
Force diagrams for different directions of motion of OMPB: (**a**) forward motion; (**b**) rightward translation; (**c**) rotational motion; (**d**) diagonal motion.

Figure 10a–d respectively depict the force situations on the vessel when it is moving forward, performing a rightward translation, rotating, and moving diagonally upward. For example, in Figure 10a during the forward movement, when all four paddle wheels rotate forward, the resultant force of the generated thrust is directed forward. As a result, the OMPB will navigate along the direction of the thrust.

## 5. Experimental Results

**Figure 11:**
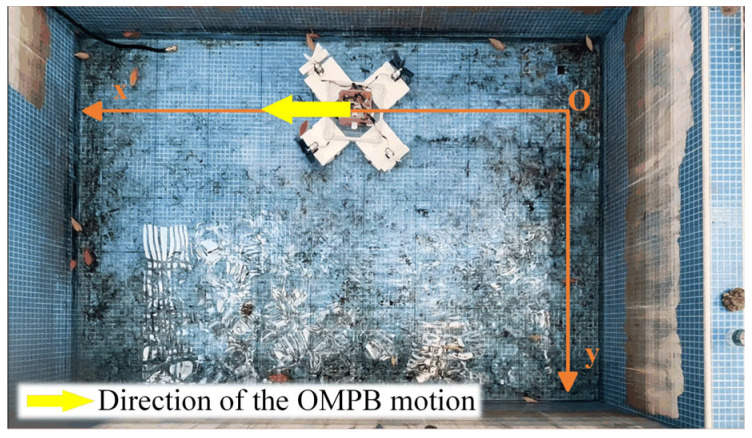
Prototype experimental testing.

To simulate narrow water areas, a water tank measuring 3 m in length and 2 m in width was constructed. To obtain the motion trajectory of the OMPB, we used the Tracker-6.2.0 software for trajectory analysis. A camera was fixed 3 m above the water tank to capture the OMPB’s movement process and record its motion trajectory at a frame rate of 30 Hz. The video of the OMPB’s trajectory was analyzed using the Tracker software, see Supplementary Material File. By establishing the center of mass of the hull to automatically track the OMPB, the position of the OMPB in each frame was found, thereby obtaining the motion trajectory of the OMPB. Due to the influence of the shooting environment and resolution, manual tracking and identifying was used for images in which it was difficult to automatically track the motion trajectory. The red electronic control box at the center of the hull was used as the center of mass position to enhance its recognition ability in image processing. The field experiment diagram is shown in Figure 11.

**Figure 12:**
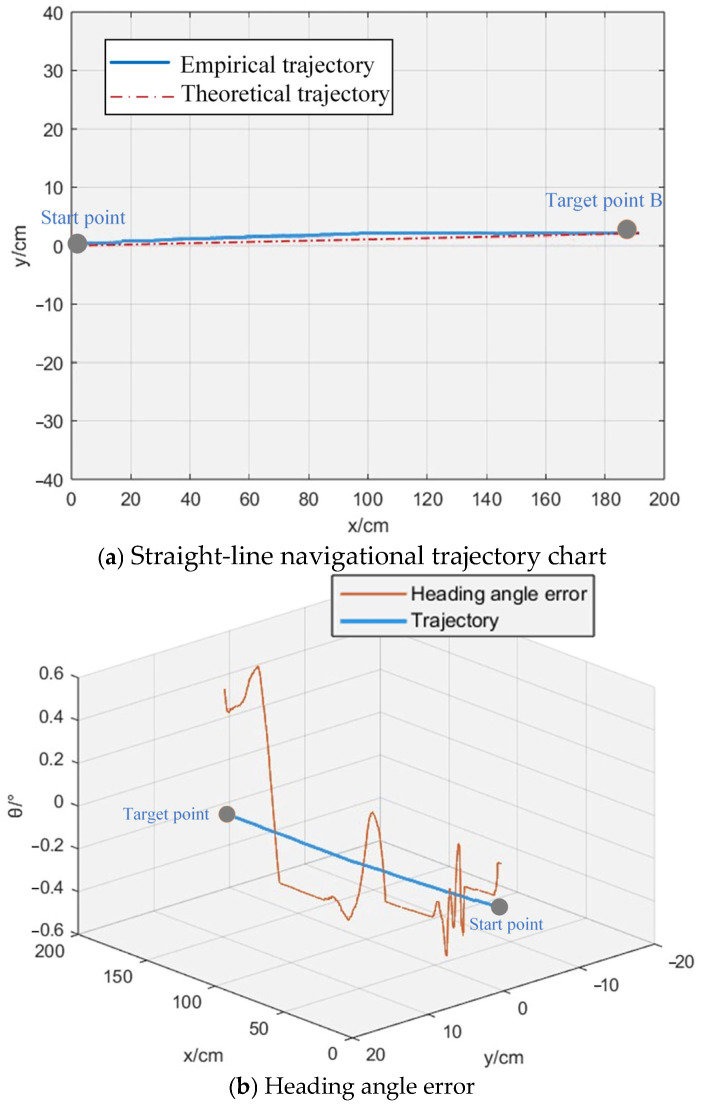
Comparison of desired trajectory and actual trajectory of straight-line navigation.

Straight-line navigation and broken-line navigation experiments were conducted. We set the rotational speed of the paddle wheels as $n_{1}=n_{2}=n_{3}=n_{4}=50\;r\cdot \min^{-1}$, and ensured that the paddle wheels had the same size dimensions. During the navigation experiments, we ensured that the OMPB maintained constant rotational speed. The actual trajectory coordinates of the OMPB during straight-line navigation were compared with the theoretical expected values, as shown in Figure 12. It can be seen that the OMPB achieves high navigation accuracy, with a trajectory tracking error within 25 mm and a yaw angle fluctuation error within 0.5°. The actual motion trajectory of the OMPB closely aligns with the expected trajectory.

**Figure 13:**
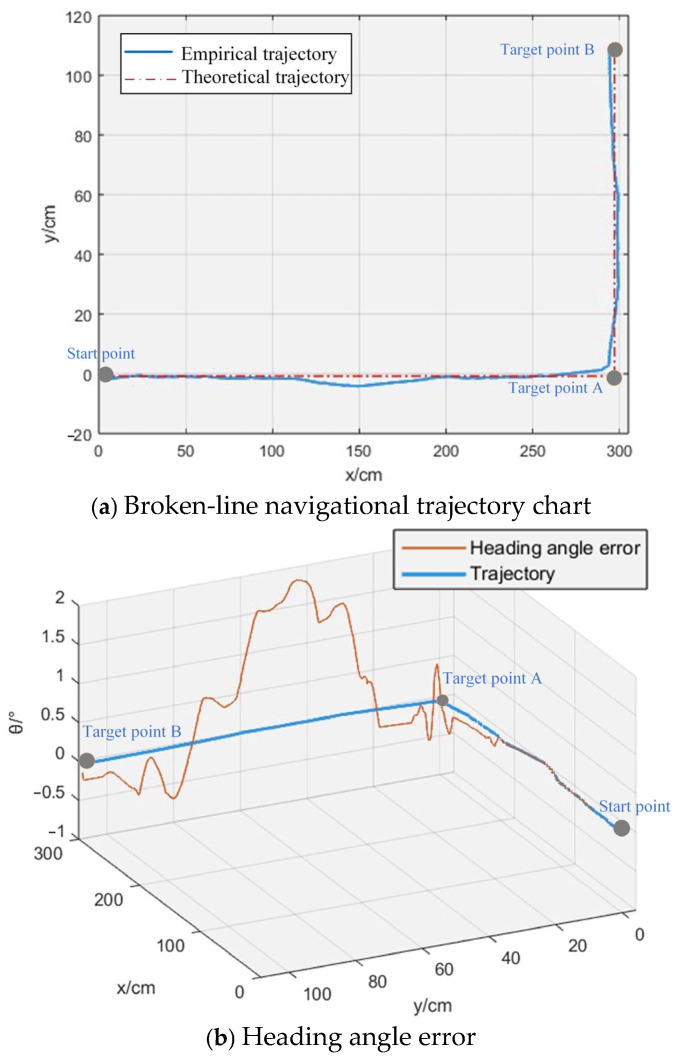
Comparison diagram between expected trajectory and actual trajectory of broken-line navigation.

The actual motion trajectory coordinates of the vessel during broken-line navigation were compared with the theoretical expected trajectory data, as shown in Figure 13. From Figure 13a, it can be observed that the actual trajectory of the broken-line navigation experiment is in good agreement with the expected trajectory, with a trajectory tracking error within 40 mm. From Figure 13b, it can be seen that as the OMPB’s operating time increases, the heading angle error reaches a value close to 2°. This is because the test was conducted in a small enclosed pool, where the waves generated by the paddle wheels’ rotation can easily perturb the OMPB’s attitude. However, the error can be gradually reduced through feedback from the IMU measurement data. Therefore, compared to the straight-line navigation experiment, the heading angle error is slightly larger, but still within 1.8°.

## 6. Conclusions

In this paper, we propose the OMPB, an omnidirectional-mobile paddle boat for operations in narrow water areas, with the advantages of omnidirectional mobility and zero turning radius. A kinematic model is established to investigate the navigation of the OMPB in water areas. This kinematic model effectively captures the relationships between the OMPB’s velocity, forces, and moments. Additionally, a fuzzy adaptive PID control is employed to control the heading of the vessel, continuously adjusting the PID parameters based on variations in the vessel’s heading, which helps to achieve trajectory tracking more precisely. Comprehensive tracking experiments demonstrate that the actual motion trajectory of the OMPB closely matches the desired trajectory, with a tracking error within 40 mm and a heading angle error within 1.8°, demonstrating excellent trajectory tracking performance and also validating the stability of the OMPB’s omnidirectional movement capability. The OMPB is characterized by its ability to steer or avoid obstacles without changing the bow direction, while it can also move laterally for berthing, which is a feature that delivers exceptionally higher efficiency when operating in narrow areas. In the future, the OMPB has potential applications in water surface garbage cleaning and fish pond feeding. In future work, we will develop practical prototypes for surface garbage removal, fish farm feed delivery, and other relevant applications.

## Figures and Tables

**Table 1:** *K_p_*, *K_i_*, *K_d_* fuzzy rules.

*E*	*E_C_*						
	NB	NM	NS	ZO	PS	PM	PB
NB	NB/ZO/PS	NB/ZO/NS	NB/ZO/NB	NM/ZO/NB	NS/ZO/NB	NS/ZO/NM	ZO/ZO/PS
NM	NB/NM/PS	NB/NM/NS	NM/NS/NB	NS/NS/NM	NS/NS/NM	ZO/ZO/NS	PS/ZO/ZO
NS	NM/NB/ZO	NM/NM/NS	NM/NS/NM	NS/NS/NM	ZO/ZO/NS	PS/PS/NS	PS/PS/ZO
ZO	NM/NB/ZO	NM/NM/NS	NS/NS/NS	ZO/ZO/NS	PS/PS/NS	PM/PM/NS	PM/PB/ZO
PS	NS/NS/ZO	NS/NS/ZO	ZO/ZO/ZO	PS/PS/ZO	PS/PS/ZO	PM/PM/ZO	PM/PB/ZO
PM	NS/ZO/PB	ZO/ZO/NS	PS/PS/PS	PM/PS/PS	PM/PM/PS	PM/PM/PS	PB/PM/PB
PB	ZO/ZO/PB	PS/ZO/PM	PM/ZO/PM	PM/ZO/PM	PM/ZO/PS	PM/ZO/PS	PB/ZO/PB

## Data Availability

Data are contained within the article and Supplementary Materials.

## Supplementary Materials

The following supporting information can be downloaded at: https://www.mdpi.com/article/10.3390/s26030866/s1, Video S1: Broken-line navigational trajectory; Video S2: Straight-line navigational trajectory.

## Abbreviations

The following abbreviations are used in this article:

OMPB	Omnidirectional-Mobile Paddle Boat
IMU	Inertial Measurement Unit
PID	Proportional–Integral–Derivative
USV	Unmanned Surface Vehicle
DC	Direct Current
