# Supplementary figures and images for: OMPB: An Omnidirectional-Mobile Paddle Boat Designed for Narrow Water Areas
Source: Sensors (Basel). 2026 Jan 28;26(3):866. doi: 10.3390/s26030866 (PMC12899551; doi:10.3390/s26030866)

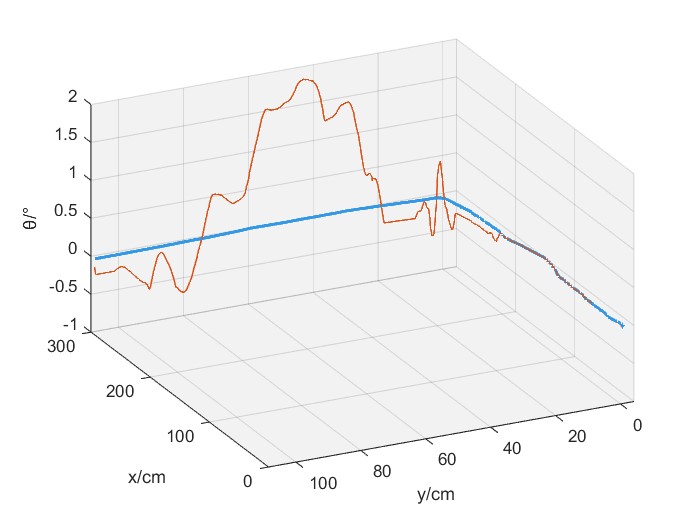

Supplement: Supplementary file 1 [file sensors-26-00866-s001.zip › Experiment_Data_sensors-404106/01_Broken-line navigational trajectory/angle_3d.jpg]

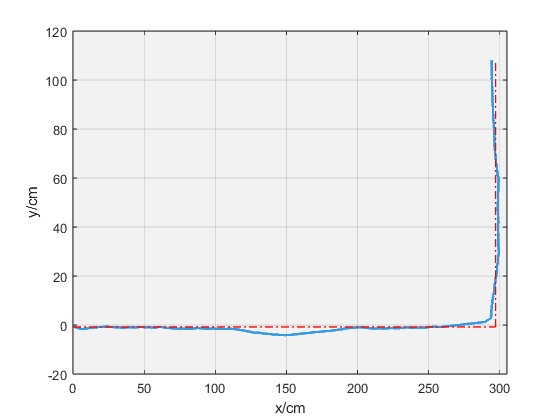

Supplement: Supplementary file 1 [file sensors-26-00866-s001.zip › Experiment_Data_sensors-404106/01_Broken-line navigational trajectory/broken_line_track.jpg]

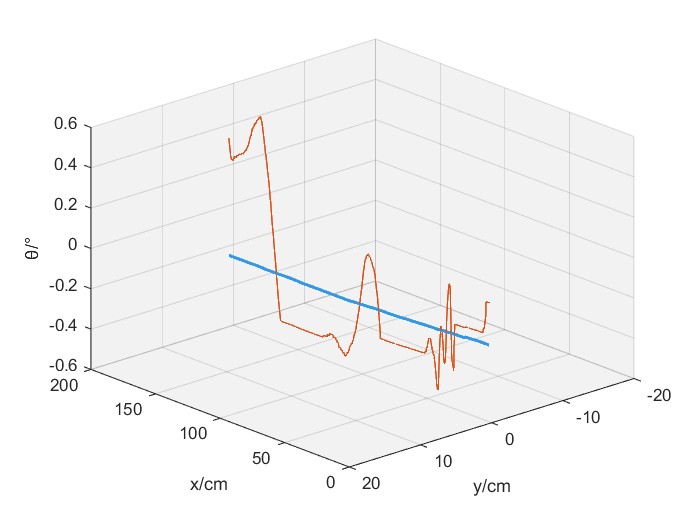

Supplement: Supplementary file 1 [file sensors-26-00866-s001.zip › Experiment_Data_sensors-404106/02_Straight-line navigational trajectory/angle_3d.jpg]

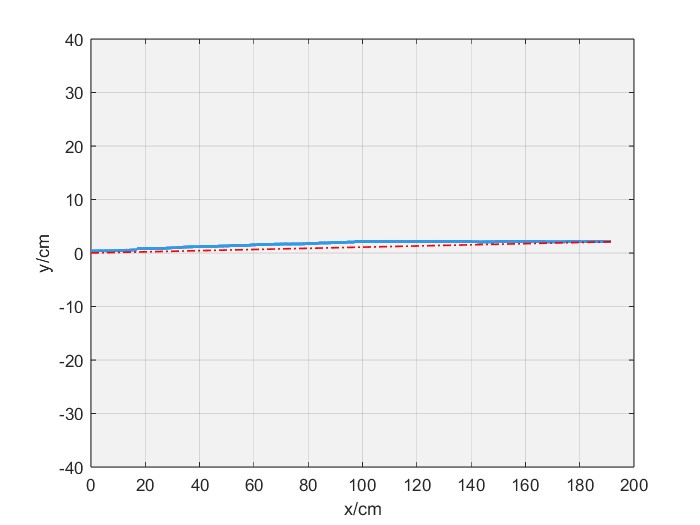

Supplement: Supplementary file 1 [file sensors-26-00866-s001.zip › Experiment_Data_sensors-404106/02_Straight-line navigational trajectory/straight_track.jpg]
